# Polyproline Peptide Aggregation with Klebsiella pneumoniae Extracellular Polysaccharides Exposes Biofilm Associated Bacteria

**DOI:** 10.1128/spectrum.02027-21

**Published:** 2022-03-07

**Authors:** Renee M. Fleeman, Bryan W. Davies

**Affiliations:** a Department of Molecular Biosciences, The University of Texas at Austin, Austin, Texas, USA; b Center for Systems and Synthetic Biology, The University of Texas at Austin, Austin, Texas, USA; c John Ring LaMontagne Center for Infectious Diseases, The University of Texas at Austin, Austin, Texas, USA; Institut Pasteur

**Keywords:** *Klebsiella pneumoniae*, polyproline peptide, biofilm, capsule, exopolysaccharide, biofilms, polyproline peptides

## Abstract

Klebsiella pneumoniae produces a thick capsule layer composed of extracellular polysaccharides protecting the bacterial cells from clearance by innate host immunity during infection. Here we characterize the interactions of a structurally diverse set of host defense peptides with K. pneumoniae extracellular polysaccharides. Remarkably, we found that all host defense peptides were active against a diverse set of K. pneumoniae strains, including hypermucoviscous strains with extensive capsule production, and aggregated with extracted capsule. Interestingly, the polyproline peptide bac7 (1-35), was the most potent antimicrobial and induced the most capsule aggregation. In addition to capsule aggregation, we found that bac7 (1-35) could also disrupt pre-formed hypermucoviscous K. pneumoniae biofilm. Further analysis using scanning electron microscopy revealed the biofilm matrix of a hypermucoviscous strain is removed by bac7 (1-35) exposing associated bacterial cells. This is the first description of a host defense peptide interacting with capsular and biofilm extracellular polysaccharides to expose cells from a K. pneumoniae biofilm matrix and suggests that features of polyproline peptides may be uniquely suited for extracellular polysaccharide interactions.

**IMPORTANCE** Klebsiella pneumoniae bacterial infections are a major threat to human health as mortality rates are steadily on the rise. A defining characteristic of K. pneumoniae is the robust polysaccharide capsule that aids in resistance to the human immune system. We have previously discovered that a synthetic peptide could aggregate with capsule polysaccharides and disrupt the capsule of K. pneumoniae. Here we describe that host defense peptides also aggregate with capsule produced from hypermucoviscous K. pneumoniae, revealing this mechanism is shared by natural peptides. We found the polyproline peptide bac7 (1-35) had the greatest antimicrobial activity and caused the most capsule aggregation. Interestingly, bac7 (1-35) also removed the biofilm matrix of hypermucoviscous K. pneumoniae exposing the associated bacterial cells. This is the first description of a polyproline peptide interacting with capsular and biofilm polysaccharides to expose cells from a K. pneumoniae biofilm matrix.

## INTRODUCTION

Klebsiella pneumoniae infections have become increasingly fatal in recent years because of the spread of multidrug resistance (1–7). This species is defined by a robust extracellular polysaccharide capsule that aids in bacterial resistance toward host immune clearance (1). Recently, hypermucoviscous strains with an increased abundance of capsule have been causing community-acquired invasive infections resulting in liver abscesses (8, 9). In addition, the thick extracellular biofilm matrix created by K. pneumoniae resists penetration by therapeutics, exacerbating the problematic infections caused by these strains (10). These factors have contributed to the increased mortality of hypermucoviscous K. pneumoniae infections (11, 12).

We recently described the evolution of a short synthetic alpha helical peptide to obtain antimicrobial activity toward capsulated K. pneumoniae (13). We found that the active peptide bound to capsule polysaccharides, causing peptide:capsule aggregates and disruption of the robust capsule layer of a hypermucoviscous K. pneumoniae strain. These findings revealed peptide binding of extracellular polysaccharides could be a mechanism that peptides use to act against invasive K. pneumoniae infections rather than a route to their inactivation.

Here we describe the investigation of a structurally diverse set of host defense peptides and their interactions with the extracellular polysaccharides of K. pneumoniae. We found that active host defense peptides aggregate with bacterial capsule polysaccharides like the synthetic sequence we previously developed. Interestingly, we found that biofilm from hypermucoviscous strains was susceptible to disruption by the polyproline peptide bac7 (1-35) (1, 2). Scanning electron microscopy of K. pneumoniae NTUH K2044 biofilms revealed that treatment with bac7 (1-35) disrupted the associated matrix material, exposing the biofilm associated bacterial cells.

## RESULTS

### Host defense peptides are active against hypermucovisous strains.

We previously described the evolution of an inactive synthetic alpha helical peptide to acquire antimicrobial activity against K. pneumoniae (13). The robust capsule of hypermucoviscous strains has been shown to increase the virulence and resistance to antimicrobial peptides (4, 14, 15). However, we found our synthetic peptide had similar activity toward hypermucoviscous and nonhypermucovisous K. pneumoniae. To determine if a similar effect occurred with naturally occurring sequences, we tested the activity of a set of host defense peptides against a panel of K. pneumoniae strains with varying capsule type.

We chose four host defense peptides with different secondary structures and host origins (Table 1) and identified the MICs of each peptide toward strains with different capsule serotypes. We tested two hypermucoviscous strains with increased capsule (NTUH K2044 and ATCC 43816), two classical nonhypermucoviscous K. pneumoniae strains (ATCC 700603 and ATCC13883), and a capsule transposon mutant *wza::180T_30_* next to its K. pneumoniae MKP103 parental strain (Table 2) (16). Previous studies suggested that increased capsule production would lead to increased antimicrobial peptide resistance (1, 2, 6, 14). However, this was not observed when we tested the antimicrobial activity of these peptides. We found indolicidin was similarly active, against the K1 hypermucoviscous strain NTUH K2044, the K2 hypermucoviscous strain 43816, and the nonhypermucoviscous type strain ATCC 13883 (Table 2). Interestingly, indolicidin had no activity toward the K. pneumoniae MKP103 parent or capsule mutant at the concentrations tested. The human cathelicidin alpha helical peptide ll-37 showed decreased antimicrobial activity toward the K1 capsule serotype hypermucoviscous strain NTUH K2044 (30 μM) compared to the nonhypermucoviscous strains (<14 μM) but was most active toward the hypermucoviscous strain K2 capsule serotype strain ATCC 43816 (2 μM). The more active peptides, beta sheet protegrin-1 and polyproline bac7 (1-35), displayed potent activity toward all strains tested with MICs of <6μM and <0.25 μM, respectively. Overall, we saw that the hypermucoviscous phenotype did not increase resistance to these peptides. Specifically, protegrin-1 had the same MIC (1.4 μM) for both hypermucoviscous strains (K1 and K2) and the nonhypermucoviscous K3 and K6 strains. In addition, bac7 (1-35) had an MIC of 0.06 μM toward the hypermucoviscous strains (K1 and K2) but variable activity toward K3 (0.03 μM) and K6 (0.12 μM) nonhypermucoviscous strains. Collectively, the spectrum of activity for these host defense peptides tested indicate that the activity is driven more by the peptide than the capsule.

**TABLE 1:** Host defense peptides^*a*^

Peptide	Sequence	MW (g/mol)	Structure	Charge	Origin	pI	# AA
Indolicidin	ILPWKWPWWPWRR - NH2	1906.28	Extended	+4	Bovine	14	13
LL-37	LLGDFFRKSKEKIGKEFKRIVQRIKDFLRNLVPRTES	4492.34	Alpha helix	+6	Human	11.6	37
Bac7 (1-35)	RRIRPRPPRLPRPRPRPLPFPRPGPRPIPRPLPFP-NH2	4206.16	Polyproline	+11	Bovine	14	35
Protegrin-1	RGGRLCYCRRRFCVCVGR - NH2 (disulfide bridge:6 - 15 and 8 - 13)	2003.48	Beta sheet	+6	Porcine	11.0	17

a MW, molecular weight; #AA, amino acids; pI, isolelectric point.

**TABLE 2:** MICs and 50% minimal biofilm eradication concentrations (MBEC_50_) of host defense peptides toward K. pneumoniae strains

μg mL^−1^/μM	Hypermucoviscous		Nonhypermucoviscous			Hypomucoviscous
	NTUH-K2044 (K1 capsule)	ATCC 43816 (K2 capsule)	ATCC 13883 (K3 capsule)	ATCC 700603 (K6 capsule)	MKP103 (K107 capsule)	MKP103 *wza* mutant
Indolicidin						
MIC	32/18	16/8	32/18	16/9	>128/>67	>128/>67
MBEC_50_	>64/>33	>64/>33	>64/>33	>64/>33	>64/>33	>64/>33
LL-37						
MIC	128/30	8/2	32/7	16/4	64/14	32/7
MBEC_50_	>64/>15	>64/>15	>64/>15	>64/>15	>64/>15	>64/>15
Bac7 (1-35)						
MIC	0.25/0.06	0.25/0.06	0.125/0.03	0.5/0.12	1/0.24	0.5/0.12
MBEC_50_	8/2	8/2	>64/>15	16/4	4/1	16/4
Protegrin-1						
MIC	0.5/1.4	0.5/1.4	0.5/1.4	0.5/1.4	2/5.6	1/2.8
MBEC_50_	32/16	8/4	>64/>30	>64/>30	4/2	32/16

### Active peptides aggregate with capsule and decrease peptide secondary structure.

**FIG 1:**
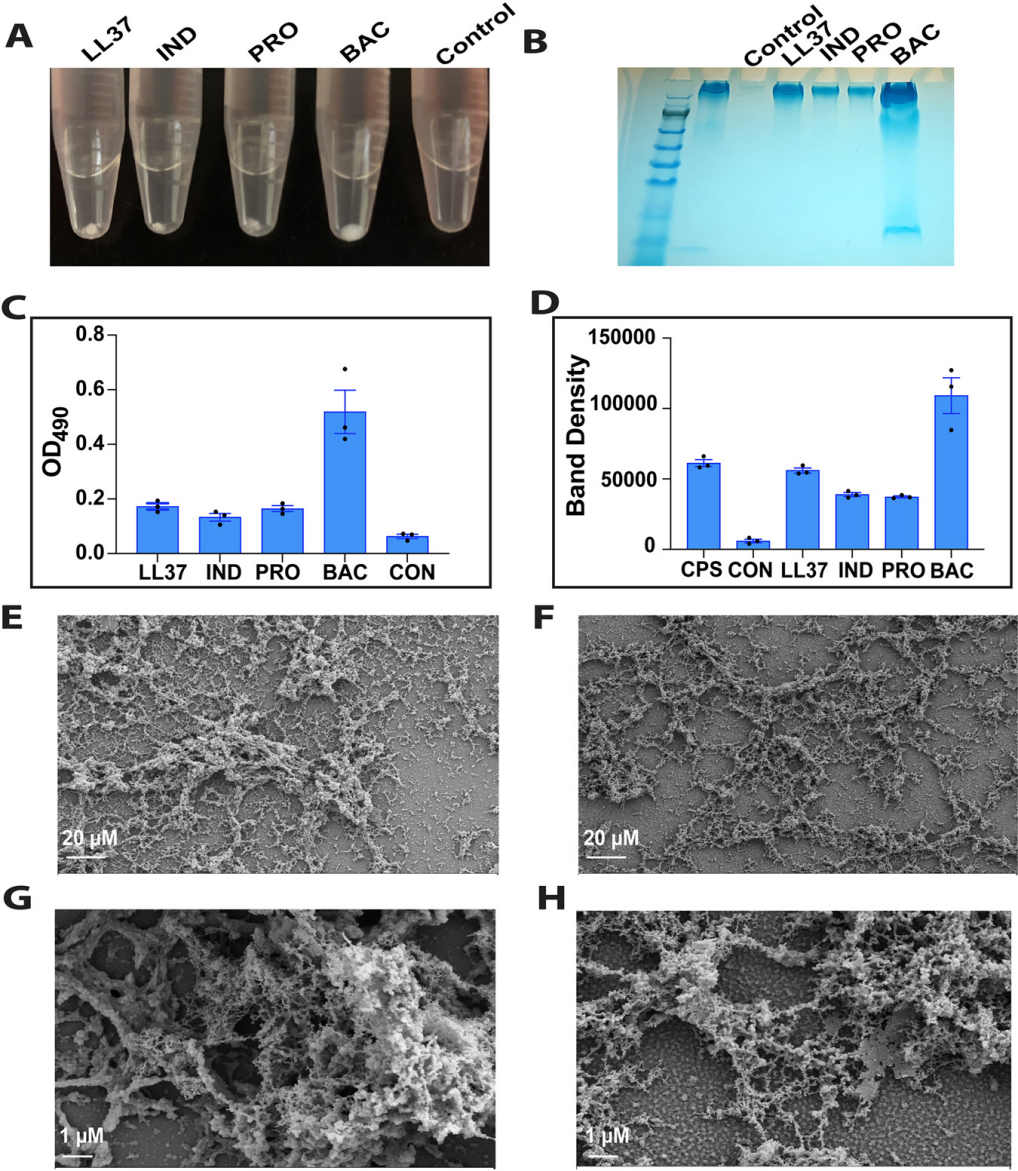
Host defense peptides aggregate with extracted capsule polysaccharides. The figure shows the exogenous capsule aggregation with host defense peptides. Purified K. pneumoniae NTUH K2044 capsule polysaccharide (200 μg mL^−1^) was assessed alone (control) or with 100 μM each peptide and centrifuged to identify aggregation pellets (A). The control and aggregates were analyzed on a 4–12% Bis-Tris gel alongside a NTUH K2044 capsule extract added at 200 μg mL^−1^ (CPS) and stained with Alcian blue to visualize polysaccharides (Fig. 1B). C shows the OD_490_ of the aggregates using uronic acid analysis to show relative amounts of polysaccharide in each aggregate. This analysis revealed bac7 (1-35) had significantly more polysaccharide than the control and all other peptide aggregates with adjusted *P* values of <0.0001 and ≤0.0004, respectively. D is the graphed band densities from the SDS page gel using ImageJ software showing bac7 (1-35) aggregate band is significantly denser compared to the aggregates from other peptides with adjusted *P* values of 0.0002, <0.0001, and <0.0001 for ll-37, indolicidin, and protegrin-1, respectively. C and D were graphed with error displayed as ±SEM and statistical significance determined using an ordinary one-way ANOVA with Tukey’s multiple comparison analysis to identify the corrected *P* values. The effect of the peptide interaction on capsule structure was analyzed using scanning electron microscopy to image 200 μg mL^−1^ of purified polysaccharide alone (E and G) or with the addition of 100 μM bac7 (1-35) (F and H).

In our previous study we showed that our active synthetic peptide aggregated with K. pneumoniae capsule, while our inactive peptide did not. Furthermore, our active synthetic peptide lost secondary structure in the presence of capsule, while our inactive peptide gained structure (13). Increase in secondary structure in the presence of extracellular polysaccharides has been described for host defense peptides previously (2, 10). Considering these observations, we sought to determine if the host defense peptides investigated here interact with extracted capsule and how this effected their secondary structures. Since all host defense peptides were active toward the hypermucoviscous strains (Table 2), and increased capsule production provides hypermucoviscous strains resistance to the host immune system, we investigated aggregation and peptide structure with capsule extracted from K. pneumoniae NTUH K2044 (K1 serotype) (Fig. 1).

**FIG 2:**
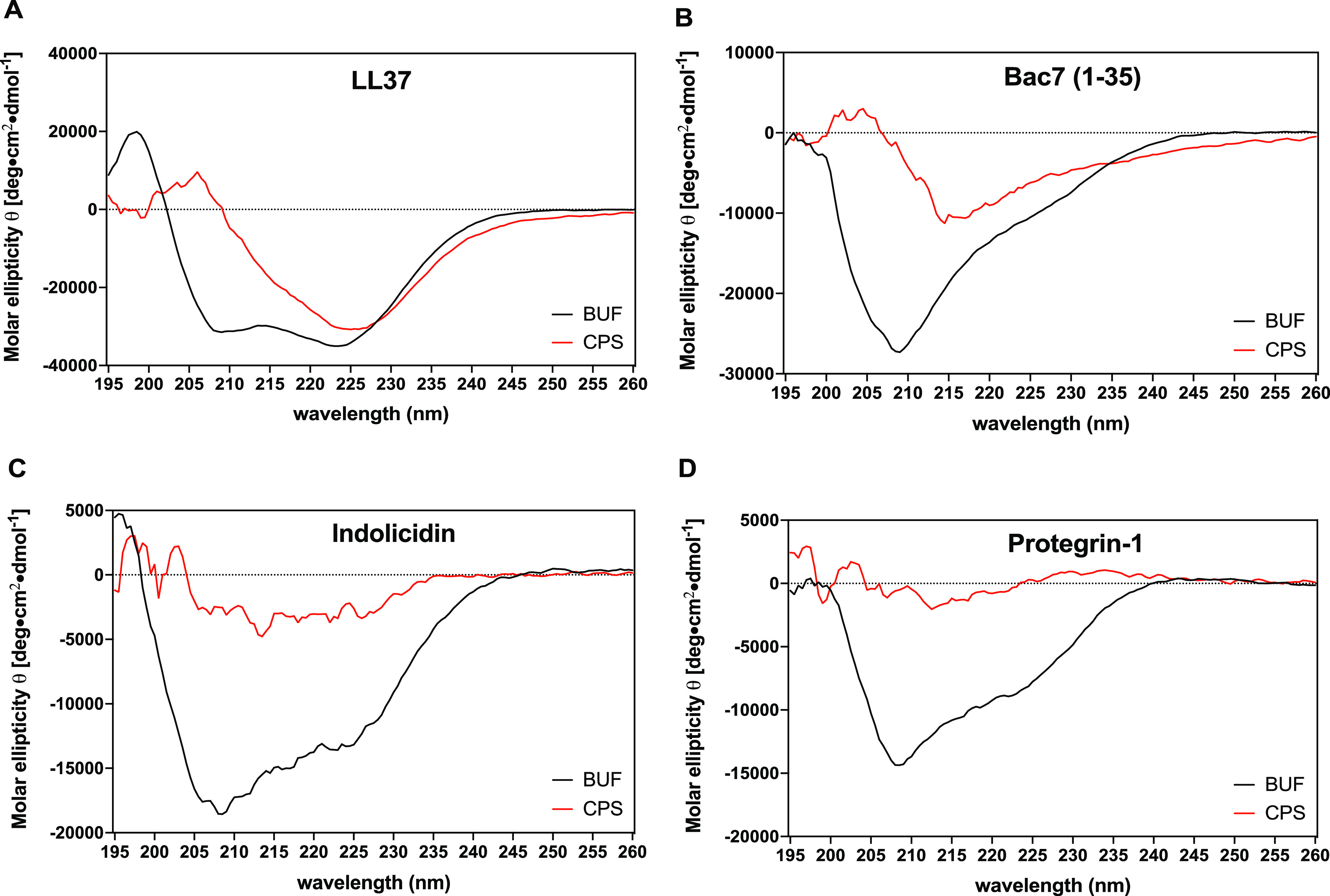
Aggregation of capsule is associated with loss of peptide structure. The figure shows the molar ellipticity CD spectra of host defense peptides (100 μM) with and without 200 μg mL^−1^ capsule extract. The spectral changes are shown for ll-37 (A), bac7 (1-35) (B), indolicidin (C), and protegrin-1 (D).

We found that all host defense peptides aggregated with capsule polysaccharides (Fig. 1A). Interestingly, the most active peptide, bac7 (1-35), did not produce a white dense pellet like the other peptides but a translucent pellet that was easier to resuspend. Analyzing these aggregates on an SDS page gel and quantifying the polysaccharide within the aggregates using a uronic acid analysis revealed that the aggregates had similar amount of capsule polysaccharide, except for bac7 (1-35), where there was a significant increase in polysaccharide within the aggregate (Fig. 1B–1D). To understand the effects of peptide treatment on the structure of capsule polysaccharide, we added bac7 (1-35) to our purified polysaccharide without centrifugation and examined the capsule polysaccharide structure with and without peptide using scanning electron microscopy (Fig. 1E–1H). With the addition of 100 μM peptide (Fig. 1F and H), the purified polysaccharide appeared thinned compared to the untreated polysaccharide (Fig. 1E and 1G). We then assessed how the peptide structures change following capsule interaction using circular dichroism and found the host defense peptides lost secondary structure with addition of capsule to varying degrees (Fig. 2). Specifically, the peptides lost their respective minima spectra present in buffer alone. We conclude that active peptides can aggregate hypermucoviscous K. pneumoniae capsule and appear to thin the complex polysaccharide structure. These polysaccharide interactions are associated with loss of peptide structure.

### K. pneumoniae biofilm increases resistance toward host defense peptides.

In addition to capsular structure, extracellular polysaccharides are also major components of the biofilm matrix (17). Biofilm matrix associated polysaccharides aid in surface attachment, protect from opsonic phagocytosis, and decrease the penetration of antimicrobials (17, 18). Although hypermucoviscous strains are typically susceptible to common antibiotics, biofilm formation can increase resistance in these strains up to 1,000 times more than planktonic state (18). Therefore, we wanted to test if the polysaccharide aggregation ability of the host defense peptides would allow for disruption of preformed K. pneumoniae biofilms.

**FIG 3:**
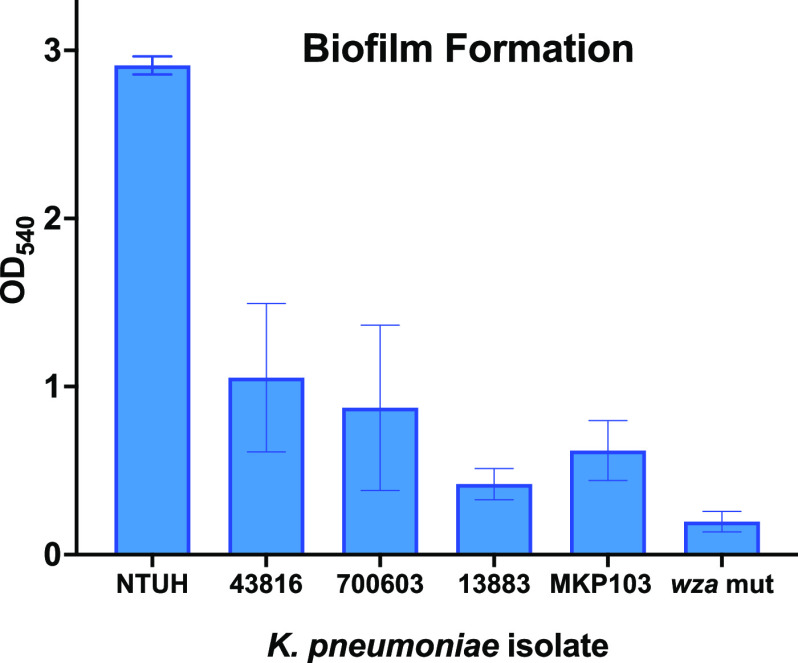
Biofilm formation using crystal violet staining. Biofilm formation abilities of K. pneumoniae NTUH K2044 (K1 serotype), 43816 (K2 serotype), ATCC 700603 (K6 serotype), and ATCC 13833 (K3 serotype), transposon parental strain MKP103, and the capsule mutant *wza::180T_30_* (*wza*). The OD_540_ values were corrected for background staining by subtracting the staining of wells with no bacteria. Error between replicates (*n* = 6) is shown as ±SEM. K. pneumoniae NTUH was found to have significantly formed more biofilm than all other strains with adjusted *P* values of <0.0001 for all strains calculated using an ordinary one-way ANOVA with Tukey’s multiple comparisons correction.

**FIG 4:**
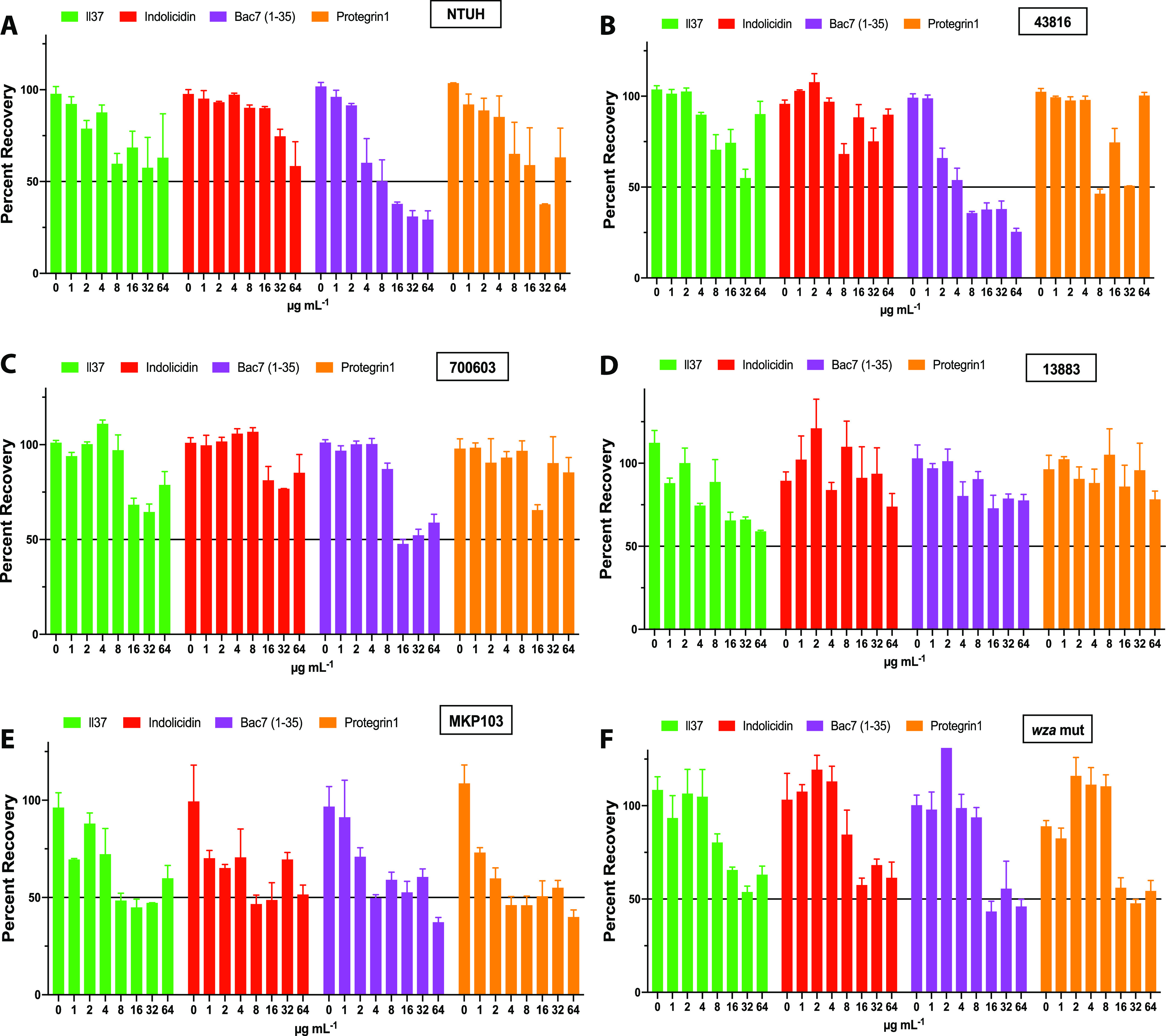
K. pneumoniae biofilm increases the resistance toward the host defense peptides. The figures below show the percent recovery of biofilm recovered following treatment with increasing concentrations (0 μg mL^−1^ – 64 μg mL^−1^) of host defense peptides for all strains in our K. pneumoniae panel. A and B show the hypermucovisous strains biofilm recovery while C and D show biofilm recovery from nonhypermucoviscous strains. E is the parental MKP103 strain, and F shows biofilm recovery of capsule mutant.

We first tested the biofilm formation of the strains used in this study (Fig. 3). The hypermucoviscous strain K. pneumoniae NTUH K2044 produced the most biofilm, and the capsule mutant *wza::180T_30_* produced the least, indicating a positive influence of polysaccharides in biofilm formation. We then tested the host defense peptides from 64 μg mL^−1^ to 1 μg mL^−1^ using 2-fold dilutions for the ability to eradicate a preformed biofilm from each of the K. pneumoniae strains (Fig. 4). Interestingly, we found that the hypermucoviscous strains, including K. pneumoniae NTUH K2044 (K1 capsule serotype), which formed the greatest amount of biofilm (Fig. 3), were more susceptible to host defense peptide biofilm eradication than nonhypermucoviscous strains (Fig. 4). Specifically, bac7 (1-35) and protegrin-1 displayed greater eradication of a preformed biofilm of strains with hypermucoviscous K1 and K2 capsule serotypes (NTUH K2044 and ATCC 43826) (Fig. 4A and B), than biofilms formed by nonhypermucoviscous strains with K3 and K6 capsule serotypes (ATCC 13883 and ATCC 700603) (Fig. 4C and 4D). Furthermore, the MKP103 *wza* capsule mutant had less biofilm formation but was less sensitive to biofilm eradication by the host defense peptides than the parental strain (ST258; K107 capsule serotype) (Fig. 3; Fig. 4E and F) (19).

Our analysis revealed more peptide was needed to eradicate a preformed biofilm than was necessary to inhibit bacterial growth in a planktonic state for all strains tested. Our results show the peptides did not completely eradicate the preformed biofilms within the concentrations tested. This could be due to the peptide solubility in this environment because in some instances, higher concentrations of the peptides resulted in less eradication. Therefore, we used the percent recovery data to calculate the 50% minimal biofilm eradication concentrations (MBEC_50_) for peptides that eradicated preformed biofilms by 50% within the concentrations tested (Table 2). LL-37 and indolicidin displayed the least biofilm eradication ability and although they did partially disrupt biofilms, we were unable to calculate MBEC_50_ values from the concentrations tested. Protegrin-1 had MBEC_50_s of 16 μM and 4 μM respectively for hypermucoviscous biofilms formed by K. pneumoniae NTUH K2044 and ATCC 43816 (Table 2). Bac7 (1-35) eradicated the biofilms of hypermucovisous strains more efficiently than any other host defense peptide with MBEC_50_s of 2 μM (Table 2). Overall, Bac7 (1-35) proved to be the most effective at eradicating biofilm of the strains tested, and remarkably performed best against hypermucoviscous strains, including K. pneumoniae NTUH K2044, which produced the greatest amount of biofilm.

### Bac7 (1-35) alters hypermucoviscous biofilm formation.

Our results show that bac7 (1-35) can weaken the biofilm created by hypermucoviscous K. pneumoniae NTUH K2044 more than biofilms from strains that produce less capsule polysaccharide. To investigate this observation, we used scanning electron microscopy (SEM) to visualize the biofilm structure of strains with different amounts of capsule polysaccharide and determine the impact of bac7 (1-35) on the structure of the biofilm matrices. The biofilms from hypermucoviscous NTUH K2044, nonhypermucoviscous strain MKP103, and the MKP103 capsule mutant were visualized with and without the addition of bac7 (1-35).

**FIG 5:**
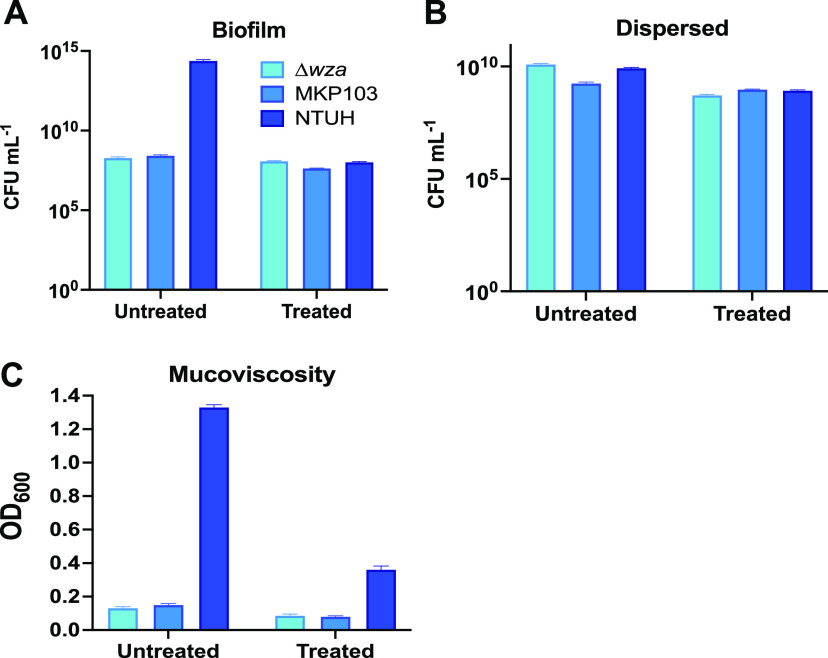
Bac7 (1-35) decreases the biofilm cell viability and mucoid phenotype of hypermucoviscous K. pneumoniae cells. The figure shows the enumeration of the cells contained within the biofilm formed on ACLAR by hypermucoviscous K. pneumoniae NTUH K2044, classical nonhypermucoviscous K. pneumoniae MKP103, and its *wza* capsule mutant (A) along with the dispersed population found within the media the biofilm was grown in (B). (C) shows the mucoviscosity of the media from all strains with and without bac7 (1-35) treatment. Error between replicates (*n* = 3) is shown as ±SEM.

The eradication assays described above (Fig. 4) grew biofilm on polyvinyl 96-well plates. For SEM studies, biofilms were grown on ACLAR film, which is commonly used for this type of analysis. In both cases, biofilms were grown overnight. The growth medium was then replaced +/- bac7 (1-35), and biofilms were incubated for another 24 h to observe bac 7 (1-35) effects. Our results in Fig. 4 showed that at 64 μM bac7 (1-35) reduced biofilm but did not completely eliminate it. We chose to use this concentration of bac7 (1-35) to ensure changes in biofilm structure could be observed. To confirm this concentration did not fully eliminate all bacteria, we assessed the viability of the biofilm associated K. pneumoniae cells grown on the ACLAR film after bac7 (1-35) (Fig. 5). Without treatment, the biofilm of hypermucoviscous K. pneumoniae NTUH K2044 contained a greater number of cells than the other strains tested. After treatment, the biofilms from all strains showed similar numbers of viable bacteria demonstrating bac7 (1-35) did not eliminate the population. Interestingly, the K. pneumoniae NTUH K2044 biofilm associated cells were more susceptible to bac7 (1-35) treatment compared to the other two strains (Fig. 5A). The increased sensitivity of K. pneumoniae NTUH K2044 biofilm to bac7 (1-35) was also observed in our biofilm eradication assay (Fig. 4). We then assessed the bacteria dispersed in the media used to grow the biofilms on ACLAR film. Following bac7 (1-35) treatment, all strains showed similar viability of dispersed bacteria and did not vary greatly from pretreatment. (Fig. 5B). Interestingly, we did find the dispersed bacteria from K. pneumoniae NTUH K2044 biofilm displayed a loss of the mucoid phenotype characteristic for this strain following treatment with bac7 (1-35) (Fig. 5C) (20, 21). These results indicate that our biofilms grown for SEM analysis display similar characteristics as biofilms grown for our eradication assays and that all biofilms and dispersed populations contain viable cells at the bac7 (1-35) concentration used.

**FIG 6:**
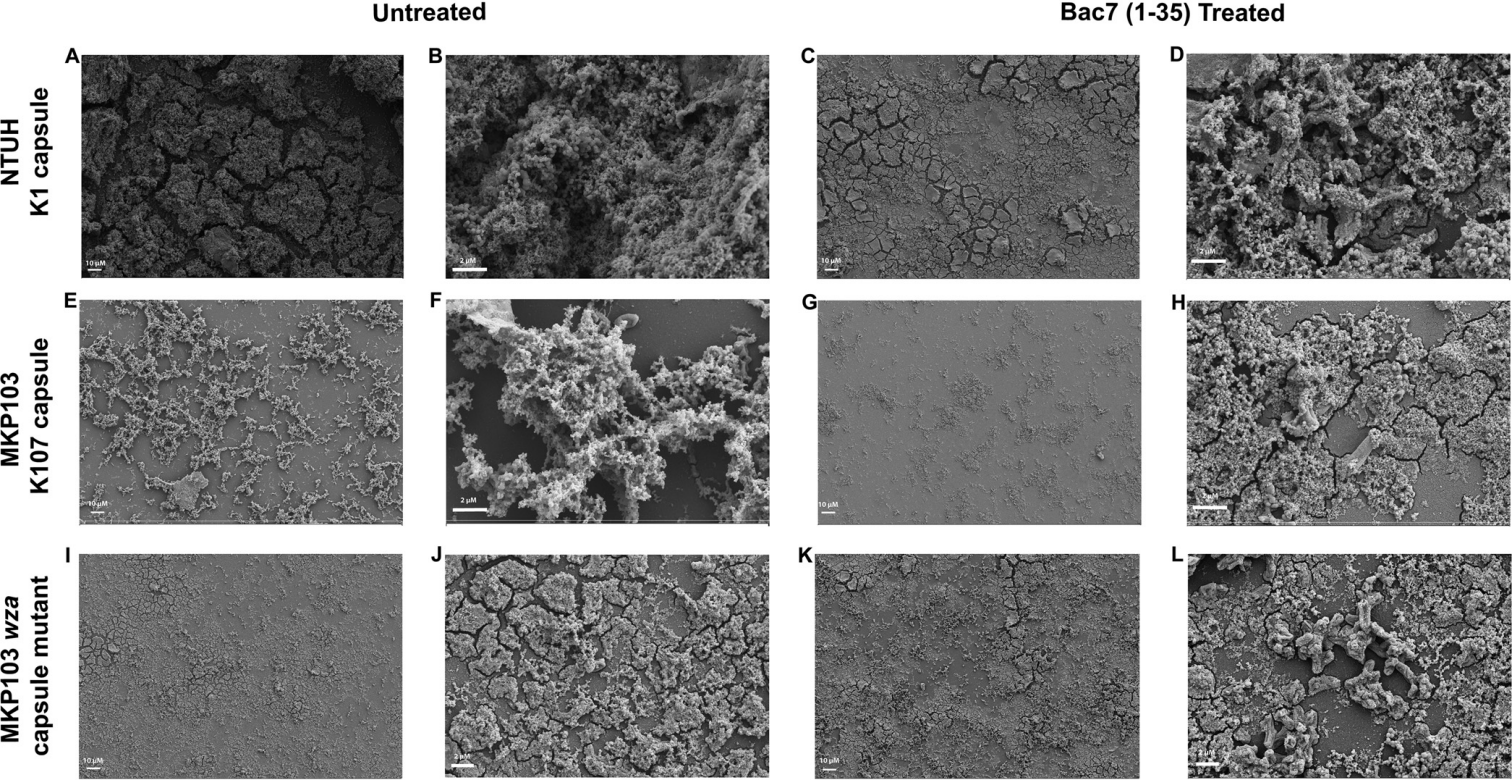
Bac7 (1-35) alters K. pneumoniae biofilm formation. The figures show SEM imaging of biofilms from hypermucoviscous K. pneumoniae NTUH K2044, classical nonhypermucoviscous K. pneumoniae MKP103, and its *wza* capsule mutant. Images in A, E, and I are the biofilms of the respective species untreated at low magnification (500×), and the images in Fig. C, G, and K are biofilms of the respective species treated with 64 μM bac7 (1-35) at low magnification (500×). The untreated biofilms (B, F, and J) and treated biofilms (D, H, and L) were imaged using higher magnification (B, D, F, and H at 6,000× or 6kx; J and L at 4kx) to visualize the detailed structures of the biofilms.

We used ruthenium red dye with tannic acid to preserve the polysaccharide component of the biofilm matrix and allow high resolution imaging (22, 23). Unlike other microscopic techniques, this approach can preserve the delicate polysaccharide component of the matrix we wish to observe. Visualizing the biofilms of all strains using SEM, we found that without treatment the biofilm of hypermucoviscous K. pneumoniae NTUH K2044 (Fig. 6A and B) had a greater density than the classical nonhypermucoviscous K. pneumoniae MKP103 (Fig. 6E and F) and the capsule mutant (Fig. 6I and J). Following bac7 (1-35) treatment, the biofilm of hypermucoviscous K. pneumoniae NTUH K2044 decreased in density and height (Fig. 6C and D) to appear structurally like what we observed with the capsule mutant (Fig. 6I–L). Similarly, following bac7 (1-35) treatment, the biofilm of classical nonhypermucoviscous K. pneumoniae MKP103 appears like the biofilm of its isogenic *wza* capsule mutant (Fig. 6G and H). These images suggest that bac7 (1-35) is damaging the biofilm matrix encasing hypermucovisous K. pneumoniae NTUH K2044 and the classical K. pneumoniae MKP103, but not the mutant lacking capsular polysaccharides.

### The dispersed hypermucoviscous biofilm cells are exposed following bac7 (1-35) treatment.

Dispersed cells from a biofilm influence the virulence of an infection and retain the matrix of the biofilm from which they emerged (24, 25). Given their importance in infection, we choose to observe the dispersed cell population from the biofilms grown for SEM and determine the effects of treatment with bac7 (1-35). Prior to processing the biofilms for SEM, the media was removed from each K. pneumoniae strain biofilm, and we dried a sample of the medium on a glass coverslip.

**FIG 7:**
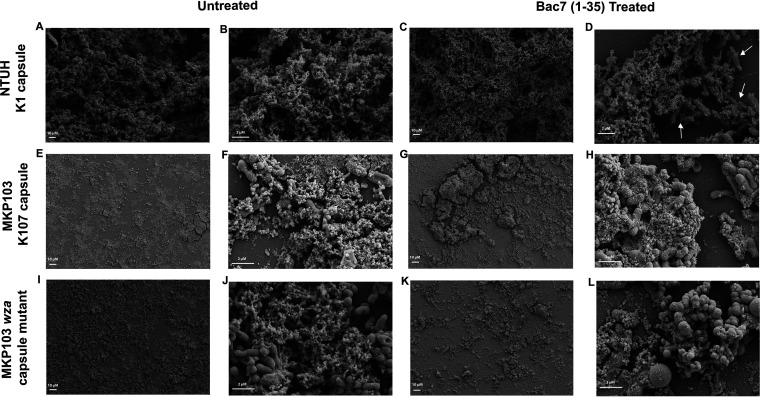
The dispersed biofilm cells are exposed with bac7 (1-35) treatment. The figures show SEM imaging of the dispersed cells from hypermucoviscous K. pneumoniae NTUH K2044, classical nonhypermucoviscous K. pneumoniae MKP103, and its *wza* capsule mutant biofilms. Images in A, E, and I are the dispersed cells of the respective species untreated at low magnification (500×), and the images in C, G, and K are biofilms of the respective species treated with 64 μM bac7 (1-35) at low magnification (500×). The untreated dispersed cells (B, F, and J) and treated dispersed cells (D, H, and L) were imaged using higher magnification (B and D at 6 kx; F, H, J, and L at 8kx) to visualize the detail of the dispersed cells and the associated matrix. White arrows on D indicate cells exposed following peptide treatment.

Under these conditions, SEM visualization revealed that hypermucoviscous K. pneumoniae NTUH K2044 dispersed cells have a large amount of matrix material (Fig. 7A and B) when compared to the other two strains (Fig. 7E, F, I, and J). Bac7 (1-35) treatment appeared to dehydrate the NTUH K2004 matrix (Fig. 7C), and increased magnification revealed individual cells (white arrows) were exposed (Fig. 7D). The effect of bac7 (1-35) was not as profound against the classical nonhypermucoviscous K. pneumoniae MKP103. In fact, we could not identify a change in the matrix of dispersed MKP103 cells following bac7 (1-35) treatment (Fig. 7G and H). The matrix surrounding the capsule mutant cells did appear slightly reduced (Fig. 7K and L), and exposed bacteria appeared rounded after bac7 (1-35) treatment, compared to their rod shape before treatment (Fig. 7J).

## DISCUSSION

K. pneumoniae hypermucoviscous strains have emerged in recent years causing infections in healthy individuals and leading to pyogenic liver abscesses (4, 8, 9, 12, 26). Here we reveal that host defense peptides have antimicrobial activity toward hypermucovisous K. pneumoniae and aggregate with extracted capsule polysaccharides. Although all peptides tested did aggregate with capsule polysaccharides, polyproline peptide bac7 (1-35) aggregates had significantly more capsule than the other host defense peptides. This may be attributed to the increased cationic nature of this peptide compared to the other host defense peptides tested, as aggregate solubility has been shown to be dependent on the cationic nature of the peptide when interacting with anionic polysaccharides (27).

Extracellular polysaccharides are not only important for capsule structure but are a major component of biofilm (28). Our study revealed that hypermucoviscous strain biofilms were susceptible to disruption by host defense peptides, with bac7 (1-35) displaying the best biofilm eradication abilities (Fig. 4, Table 2). We also found that the parental strain MKP103 produced a more robust biofilm but was more susceptible to bac7 (1-35) eradication than its capsule mutant. In addition, using SEM we observed that biofilm treatment with bac7 (1-35) had a greater effect on biofilms from strains that produce greater amount of matrix material, decreasing the volume of the biofilm (Fig. 6). This was similar to what was observed by Benincasa et al., that found treatment of K. pneumoniae biofilms with 64 μM bac7 (1-35) resulted in a reduction in biofilm height and in increase in roughness (29). Collectively, these findings suggest that bac7 (1-35) may have an ability to interact with both capsular polysaccharides and polysaccharides that compose the biofilm matrix.

Dispersed cells from K. pneumoniae biofilms are distinct from planktonically growing cells (30), and have an important role in the virulence of this species (24). Furthermore, capsule upregulation in mature biofilms may indicate an important role in capsule for dispersed cells (17). Therefore, we wanted to observe the phenotypic variations of the biofilm dispersed cells that accompany bac7 (1-35) treatment. Viewing the dispersed cells from the biofilm media using SEM, we found hypermucoviscous K. pneumoniae NTUH K2044 individual cells are more exposed following bac 7 (1-35) treatment (Fig. 7D).

Taken together, we have found that host defense peptides do aggregate with the capsule of hypermucoviscous K. pneumoniae. Importantly, bac7 (1-35) can disrupt the biofilm matrix barrier that impedes antimicrobial therapies and the host immune system. Targeting the polysaccharides of the biofilm matrix could increase the susceptibility of hypermucoviscous K. pneumoniae infections that have become problematic in recent years. Investigating interactions between bac7 (1-35) and biofilm polysaccharides and increasing our understanding of the increased susceptibility of hypermucoviscous strains will be a future focus for our lab.

## MATERIALS AND METHODS

### Bacterial strains and growth conditions.

All bacterial strains used in this study are listed in Table 3. The parental strain MKP103 and transposon mutant deficient in capsule were acquired from the Manoil lab at the University of Washington (16). All bacterial strains were grown overnight in lysogeny broth (LB) at 37°C with shaking at 225 rpm.

**TABLE 3:** Bacterial strains used in this study

K. pneumoniae	Characteristic	Isolation	Reference
NTUH K2044	K1 capsule serotype	Liver abscess	38
ATCC 43816	K2 capsule serotype	pneumonia strain	39
MKP103	ST258; K107	NIH clinical outbreak	16, 19
MKP103*wza::180T_30_*	Capsule deficient	Transposon mutant	16
ATCC 13883	Type strain; K3		40
ATCC 700603	Clinical strain; K6	Urine	41

### Peptides.

The host defense peptides ll-37, indolicidin, and protegrin-1 were ordered from Anaspec (https://www.anaspec.com). The bac7 (1-35) peptide was ordered from Novopro (https://www.novoprolabs.com). All peptides were resuspended in ultrapurified water at 10 mg mL^−1^ and stored at −20°C.

### Antibacterial assays.

All minimal inhibitory assays were performed using the broth dilution method in 96-well plates with the peptides tested in triplicate by serial diluting in 0.2% BSA 0.01% acetic acid solution (31). Bacteria were synchronized to mid-log phase and standardized to a final OD_600_ of 0.001 in Mueller-Hinton broth to yield ∼5 × 10^5^ CFU mL^−1^. The assay incubated for 20 h at 37°C before determining MICs. MICs were determined when no visible growth was present in all three replicates.

### Capsule extraction, purification, and quantification.

Following overnight incubation of K. pneumoniae NTUH K2044 in LB broth at 37°C with shaking, 500 mL was centrifuged for 15 min at 10,000 rpm and 4°C. The polysaccharide capsule was extracted using the hot phenol method (2). Briefly, the pellet was washed once with water and resuspended in 50 mL water, and 500 μL was aliquoted into microcentrifuge tubes. The suspensions were incubated for 2 min at 68°C, 500 μL of phenol was added, and the samples were incubated for 30 min at 68°C. Following incubation, 500 μL of chloroform was added and the suspension was centrifuged at 14,000 rpm for 5 min to separate and remove the aqueous layer. To precipitate the polysaccharides from the suspension, 3 volumes of ethanol were added, and the sample placed at −20°C overnight. The precipitated polysaccharides were recovered by centrifuging at 14,000 rpm for 30 min, and the ethanol subsequently removed. Following extraction, the polysaccharides were resuspended in 500 μL water and dialyzed against water overnight using a 100 Dalton dialysis membrane. Following dialysis, the samples were dried using an Eppendorf Vacufuge plus and resuspended at 10 μg mL^−1^ in 0.8% NaCl/0.05% NaN3/0.1 M Tris HCl (pH 7). The solution was then treated with 50 μg mL^−1^ of DNase II typeV and RNase A for 18 h at 37°C. This was followed by protein digestion using 50 μg mL^−1^ of Proteinase K and incubating for 1 h at 55°C followed by 24-h incubation at room temperature. The polysaccharides were then precipitated using 5 volumes of methanol with 1% vol/vol of saturated sodium acetate and incubation at −20°C overnight. Finally, the sample was resuspended in water and LPS was removed using a Beckmann Coulter ultracentrifuge at 105,000 × *g* for 20 h at 4°C and repeating this step twice. The polysaccharide extract was quantified using the uronic acid method (32). Briefly, 200 μL of the polysaccharide extract was added to glass test tubes, followed by the addition of 200 μL 5% phenol and 1 mL of 93% sulfuric acid. The solution was mixed by swirling and incubated at room temperature for 10 min before measuring the OD_490_ and comparing to a standard curve to quantify.

### Circular dichroism.

The circular dichroism performed in this study was performed using the Jasco-815 CD spectrometer at the Targeted Therapeutic Drug Discovery & Development Program Core at UT Austin. The peptides (100 μM) were diluted in 10 mM potassium phosphate buffer with and without 200 μg mL^−1^ of extracted capsule polysaccharides to analyze a 200 μL volume using a 0.1-cm path-length quartz cuvette. The CD spectra were collected using far-UV spectra (190–260 nm) with background corrected using 10 mM potassium phosphate buffer with or without added capsule. Molar ellipticity was calculated as described (33, 34). Specifically, the resulting spectra were measured ellipticity (mdeg) and converted to molar ellipticity using the formula θ = (peptide MW/n)*(mdeg)/(10*C*L), where n is number of amino acids in the peptide, C is the concentration of the peptide in mg mL^−1^, and L is the path length of the quartz cuvette.

### Aggregation assays.

The host defense peptides were added at 100 μM to extracted capsule (200 μg mL^−1^) in a phosphate buffer, vortexed, and immediately centrifuged at 14,000 rpm for 15 min. The pellets were washed 2 times with ultrapurified water, and resuspended in 200 μL ultrapurified water for quantification using the uronic acid method as described above or 20 μL ultrapurified water for SDS page analysis. Before SDS page analysis, the resuspended aggregates were boiled for 10 min at 100°C with SDS page loading dye and run on a 4–15% bis-tris SDS page gel. The controls used in SDS page analysis included (i) the no peptide control tube (CPS) sample that was treated along with the pellets to show if capsule would aggregate and retain in the tubes without peptides (Fig. 1A); and (ii) the control lane to the gel that had purified 200 μg mL^−1^ (control) to reveal how our purified capsule extract looked like on the SDS page gel (Fig. 1B). The gel was washed 6 times with ultrapurified water, stained with alcian blue for 60 min, and detained overnight with 60% 20 mM solidum acetate (pH 4.75), 40% ethanol (35). The aggregation, uronic acid, and SDS page analyses were performed in triplicate with one representative gel shown (Fig. 1B). ImageJ software was used to determine band density of the triplicate gels with the resulting values graphed with error presented as ±SEM.

### Biofilm eradication assays.

Overnight cultures of K. pneumoniae strains were diluted to an OD_600_ of 0.5 (9.75 × 10^9^) in biofilm media (tryptic soy broth, 3% NaCl, and 0.5% Glucose) and grown at 37°C without shaking for 24 h. The biofilm media recipe we used includes increased sodium and glucose, components described to increase *in vitro* formation of biofilms for K. pneumoniae (36). When assessing biofilm formation abilities and peptide eradication potential, we grew the biofilms in Corning polyvinyl chloride (PVC) 96-well round-bottom plates. The plate contained media-only wells with no bacteria that were treated with the same crystal violet staining procedure to account for the crystal violet background staining of the wells. For the biofilm eradication assays, the medium was removed from the preformed biofilm and Mueller-Hinton broth was added with the host defense peptide dilutions. Once the medium containing the peptides was carefully added to the biofilm, the plates were incubated at 37°C without shaking for 24 h. The biofilm was washed 3 times with phosphate-buffered saline and stained with 0.1% crystal violet for 10 min, then washed 3 more times before drying overnight. The stain was solubilized with 30% acetic acid and transferred to a polystyrene 96-well plate, and the optical density at 540 nm was assessed using a Spectramax Plate Reader. All biofilm testing was performed using biological triplicates. The data were graphed as optical density values for Fig. 3 with the background staining of wells subtracted and error presented as ±SEM. For Fig. 4, the data were presented as percent recovery using the no-treatment wells as 100% recovery with error presented as ±SEM.

### Mucoviscosity assay.

The biofilm media was assessed for mucoviscosity as previously described (20, 21).The media from triplicate ACLAR grown biofilms from each strain were removed and placed in a 1.5 mL microcentrifuge tube. Optical density at 600 nm was obtained before and after centrifugation at 1,000 × *g* for 5 min. The OD_600_ values obtained after centrifugation were normalized to the starting OD_600_ for K. pneumoniae NTUH K2044 untreated before centrifugation. The resulting values were graphed with error reported as ±SEM.

### Microscopy imaging.

Overnight cultures of K. pneumoniae NTUH K2044, MKP103, and MKP103*wza::180T_30_* were diluted to an OD_600_ of 0.5 in biofilm media (9.75 × 10^9^ CFU mL^−1^), and 2 mL was added to a 35 × 10 mm petri dish containing the ACLAR (Ted Pella Inc.) film and incubated without shaking at 37°C for 24 h. ACLAR is transparent, chemically inert material that is stable for use in scanning electron microscopy (37). After developing a biofilm on ACLAR film in a petri dish, we replaced the media with either Mueller-Hinton broth (MHB) with no treatment or MHB with 64 μM Bac7 (1-35) and allowed the biofilms to incubate at 37°C for 24 h. All biofilms were grown with *n* = 4 to allow for enumeration in triplicate and SEM imaging. The ACLAR film was washed with phosphate-buffered saline (PBS), placed in a 50 mL conical tube with 2 mL PBS, and vortexed to detach the biofilm. To enumerate the biofilm and dispersed cells, the suspended biofilm was serial diluted using 10-fold dilutions alongside the media containing the dispersed cells, plated in technical duplicate on LB agar, and grown overnight at 37°C. For SEM analysis, the dispersed cells were concentrated by plating 50 μL of culture on a glass coverslip, drying, and treating it with the same procedure as the biofilm samples as explained below. ACLAR disks with biofilm and glass coverslips with dispersed cells were fixed overnight in 4% glutaraldehyde and 2% paraformaldehyde with 0.15% Ruthenium Red (RR) and 1% tannic acid in 0.2 M Na Cacodylate buffer, pH 7.4. Fixed cells were gently washed in 0.15% Ruthenium red in buffer, then stained with 1% osmium tetroxide with 0.15% RR in buffer for approximately 2 h. The samples were washed gently with water, then dehydrated through graded alcohols, transferred to 1:1 absolute ethanol in hexamethyldisilazane (HDMS) for 10 min, then 5 min in HDMS, and air dried for 30 min. Samples were sputter coated with 7 nm platinum:palladium (Pt:Pd) and imaged in a Zeiss Supra SEM at 5 kV accelerating voltage.

## References

[1] Bengoechea JA, Sa Pessoa J. 2019. Klebsiella pneumoniae infection biology: living to counteract host defences. FEMS Microbiol Rev 43:123–144. doi:10.1093/femsre/fuy043. 30452654 PMC6435446

[2] Campos MA, Vargas MA, Regueiro V, Llompart CM, Alberti S, Bengoechea JA. 2004. Capsule polysaccharide mediates bacterial resistance to antimicrobial peptides. Infect Immun 72:7107–7114. doi:10.1128/IAI.72.12.7107-7114.2004. 15557634 PMC529140

[3] Gomez-Simmonds A, Uhlemann AC. 2017. Clinical implications of genomic adaptation and evolution of carbapenem-resistant *Klebsiella pneumoniae*. J Infect Dis 215:S18–S27. doi:10.1093/infdis/jiw378. 28375514 PMC5853309

[4] Li W, Sun G, Yu Y, Li N, Chen M, Jin R, Jiao Y, Wu H. 2014. Increasing occurrence of antimicrobial-resistant hypervirulent (hypermucoviscous) *Klebsiella pneumoniae* isolates in China. Clin Infect Dis 58:225–232. doi:10.1093/cid/cit675. 24099919

[5] Marr CM, Russo TA. 2019. Hypervirulent *Klebsiella pneumoniae*: a new public health threat. Expert Rev Anti Infect Ther 17:71–73. doi:10.1080/14787210.2019.1555470. 30501374 PMC6349525

[6] Martin RM, Bachman MA. 2018. Colonization, infection, and the accessory genome of *Klebsiella pneumoniae*. Front Cell Infect Microbiol 8:4. doi:10.3389/fcimb.2018.00004. 29404282 PMC5786545

[7] Meatherall BL, Gregson D, Ross T, Pitout JDD, Laupland KB. 2009. Incidence, risk factors, and outcomes of *Klebsiella pneumoniae* bacteremia. Am J Med 122:866–873. doi:10.1016/j.amjmed.2009.03.034. 19699383

[8] Patel PK, Russo TA, Karchmer AW. 2014. Hypervirulent *Klebsiella pneumoniae*. Open Forum Infect Dis 1:ofu028. doi:10.1093/ofid/ofu028. 25734101 PMC4324179

[9] Russo TA, Marr CM. 2019. Hypervirulent *Klebsiella pneumoniae*. Clin Microbiol Rev 32:e00001-19. doi:10.1128/CMR.00001-19. 31092506 PMC6589860

[10] Bellich B, Lagatolla C, Tossi A, Benincasa M, Cescutti P, Rizzo R. 2018. Influence of bacterial biofilm polysaccharide structure on interactions with antimicrobial peptides: a study on *Klebsiella pneumoniae*. Int J Mol Sci 19:1685. doi:10.3390/ijms19061685. PMC6032227 29882774

[11] Xu L, Sun X, Ma X. 2017. Systematic review and meta-analysis of mortality of patients infected with carbapenem-resistant *Klebsiella pneumoniae*. Ann Clin Microbiol Antimicrob 16:18. doi:10.1186/s12941-017-0191-3. 28356109 PMC5371217

[12] Zhang Y, Zhao C, Wang Q, Wang X, Chen H, Li H, Zhang F, Li S, Wang R, Wang H. 2016. High prevalence of hypervirulent *Klebsiella pneumoniae* infection in China: geographic distribution, clinical characteristics, and antimicrobial resistance. Antimicrob Agents Chemother 60:6115–6120. doi:10.1128/AAC.01127-16. 27480857 PMC5038323

[13] Fleeman RM, Macias LA, Brodbelt JS, Davies BW. 2020. Defining principles that influence antimicrobial peptide activity against capsulated *Klebsiella pneumoniae*. Proc Natl Acad Sci USA 117:27620–27626. doi:10.1073/pnas.2007036117. 33087568 PMC7959497

[14] Shon AS, Bajwa RPS, Russo TA. 2013. Hypervirulent (hypermucoviscous) *Klebsiella pneumoniae*. Virulence 4:107–118. doi:10.4161/viru.22718. 23302790 PMC3654609

[15] Llobet E, Tomás JM, Bengoechea JA. 2008. Capsule polysaccharide is a bacterial decoy for antimicrobial peptides. Microbiology (Reading) 154:3877–3886. doi:10.1099/mic.0.2008/022301-0. 19047754

[16] Ramage B, Erolin R, Held K, Gasper J, Weiss E, Brittnacher M, Gallagher L, Manoil C. 2017. Comprehensive arrayed transposon mutant library of *Klebsiella pneumoniae* outbreak strain KPNIH1. J Bacteriol 199:e00352-17. doi:10.1128/JB.00352-17. 28760848 PMC5637181

[17] Limoli DH, Jones CJ, Wozniak DJ. 2015. Bacterial extracellular polysaccharides in biofilm formation and function. Microbiol Spectr 3. doi:10.1128/microbiolspec.MB-0011-2014. PMC4657554 26185074

[18] Zheng J-x, Lin Z-w, Chen C, Chen Z, Lin F-j, Wu Y, Yang S-y, Sun X, Yao W-m, Li D-y, Yu Z-j, Jin J-l, Qu D, Deng Q-w. 2018. Biofilm formation in *Klebsiella pneumoniae* bacteremia strains was found to be associated with CC23 and the presence of *wcaG*. Front Cell Infect Microbiol 8:21. doi:10.3389/fcimb.2018.00021. 29527517 PMC5829044

[19] Choby JE, Howard‐Anderson J, Weiss DS. 2020. Hypervirulent *Klebsiella pneumoniae*—clinical and molecular perspectives. J Intern Med 287:283–300. doi:10.1111/joim.13007. 31677303 PMC7057273

[20] Bachman MA, Breen P, Deornellas V, Mu Q, Zhao L, Wu W, Cavalcoli JD, Mobley HLT. 2015. Genome-wide identification of *Klebsiella pneumoniae* fitness genes during lung infection. mBio 6:e00775-15. doi:10.1128/mBio.00775-15. 26060277 PMC4462621

[21] Walker KA, Miner TA, Palacios M, Trzilova D, Frederick DR, Broberg CA, Sepúlveda VE, Quinn JD, Miller VL. 2019. A *Klebsiella pneumoniae* regulatory mutant has reduced capsule expression but retains hypermucoviscosity. mBio 10:e00089-19. doi:10.1128/mBio.00089-19. 30914502 PMC6437046

[22] Erlandsen SL, Kristich CJ, Dunny GM, Wells CL. 2004. High-resolution visualization of the microbial glycocalyx with low-voltage scanning electron microscopy: dependence on cationic dyes. J Histochem Cytochem 52:1427–1435. doi:10.1369/jhc.4A6428.2004. 15505337 PMC3957825

[23] Relucenti M, Familiari G, Donfrancesco O, Taurino M, Li X, Chen R, Artini M, Papa R, Selan L. 2021. Microscopy methods for biofilm imaging: focus on SEM and VP-SEM pros and cons. Biology 10:51. doi:10.3390/biology10010051. 33445707 PMC7828176

[24] Guilhen C, Forestier C, Balestrino D. 2017. Biofilm dispersal: multiple elaborate strategies for dissemination of bacteria with unique properties. Mol Microbiol 105:188–210. doi:10.1111/mmi.13698. 28422332

[25] Rumbaugh KP, Sauer K. 2020. Biofilm dispersion. Nat Rev Microbiol 18:571–586. doi:10.1038/s41579-020-0385-0. 32533131 PMC8564779

[26] Zhang S, Zhang X, Wu Q, Zheng X, Dong G, Fang R, Zhang Y, Cao J, Zhou T. 2019. Clinical, microbiological, and molecular epidemiological characteristics of *Klebsiella pneumoniae*-induced pyogenic liver abscess in southeastern China. Antimicrob Resist Infect Control 8:166. doi:10.1186/s13756-019-0615-2. 31673355 PMC6819602

[27] Ghosh KA, Bandyopadhyay P. 2012. Polysaccharide-protein interactions and their relevance in food colloids. *In* Karunaratne DN (ed), The complex world of polysaccharides. IntechOpen, London, United Kingdom. doi:10.5772/50561.

[28] Flemming H-C, Wingender J. 2010. The biofilm matrix. Nat Rev Microbiol 8:623–633. doi:10.1038/nrmicro2415. 20676145

[29] Benincasa M, Lagatolla C, Dolzani L, Milan A, Pacor S, Liut G, Tossi A, Cescutti P, Rizzo R. 2016. Biofilms from *Klebsiella pneumoniae*: matrix polysaccharide structure and interactions with antimicrobial peptides. Microorganisms 4:26. doi:10.3390/microorganisms4030026. PMC5039586 27681920

[30] Guilhen C, Miquel S, Charbonnel N, Joseph L, Carrier G, Forestier C, Balestrino D. 2019. Colonization and immune modulation properties of *Klebsiella pneumoniae* biofilm-dispersed cells. NPJ Biofilms Microbiomes 5:25. doi:10.1038/s41522-019-0098-1. 31583108 PMC6760147

[31] Wiegand I, Hilpert K, Hancock REW. 2008. Agar and broth dilution methods to determine the minimal inhibitory concentration (MIC) of antimicrobial substances. Nat Protoc 3:163–175. doi:10.1038/nprot.2007.521. 18274517

[32] Brimacombe C, Beatty J. 2013. Surface polysaccharide extraction and quantification. Bio-Protocol 3:e934. doi:10.21769/BioProtoc.934. e934.

[33] Greenfield NJ. 2006. Using circular dichroism spectra to estimate protein secondary structure. Nat Protoc 1:2876–2890. doi:10.1038/nprot.2006.202. 17406547 PMC2728378

[34] Kelly SM, Jess TJ, Price NC. 2005. How to study proteins by circular dichroism. Biochim Biophys Acta 1751:119–139. doi:10.1016/j.bbapap.2005.06.005. 16027053

[35] Karlyshev AV, Wren BW. 2001. Detection and initial characterization of novel capsular polysaccharide among diverse *Campylobacter jejuni* strains using Alcian blue dye. J Clin Microbiol 39:279–284. doi:10.1128/JCM.39.1.279-284.2001. 11136784 PMC87715

[36] Singh AK, Yadav S, Chauhan BS, Nandy N, Singh R, Neogi K, Roy JK, Srikrishna S, Singh RK, Prakash P. 2019. Classification of clinical isolates of *Klebsiella pneumoniae* based on their *in vitro* biofilm forming capabilities and elucidation of the biofilm matrix chemistry with special reference to the protein content. Front Microbiol 10:669. doi:10.3389/fmicb.2019.00669. 31019496 PMC6458294

[37] Kingsley RE, Cole NL. 1988. Preparation of cultured mammalian cells for transmission and scanning electron microscopy using aclar film. J Electron Microsc Tech 10:77–85. doi:10.1002/jemt.1060100110. 3193245

[38] Wu K-M, Li L-H, Yan J-J, Tsao N, Liao T-L, Tsai H-C, Fung C-P, Chen H-J, Liu Y-M, Wang J-T, Fang C-T, Chang S-C, Shu H-Y, Liu T-T, Chen Y-T, Shiau Y-R, Lauderdale T-L, Su I-J, Kirby R, Tsai S-F. 2009. Genome sequencing and comparative analysis of *Klebsiella pneumoniae* NTUH-K2044, a strain causing liver abscess and meningitis. J Bacteriol 191:4492–4501. doi:10.1128/JB.00315-09. 19447910 PMC2704730

[39] Bakker-Woudenberg IA, van den Berg JC, Vree TB, Baars AM, Michel MF. 1985. Relevance of serum protein binding of cefoxitin and cefazolin to their activities against *Klebsiella pneumoniae* pneumonia in rats. Antimicrob Agents Chemother 28:654–659. doi:10.1128/AAC.28.5.654. 3911879 PMC176351

[40] Cowan ST, Steel KJ, Shaw C, Duguid JP. 1960. A classification of the Klebsiella group. J Gen Microbiol 23:601–612. doi:10.1099/00221287-23-3-601. 13696095

[41] Rasheed JK, Anderson GJ, Yigit H, Queenan AM, Domenech-Sanchez A, Swenson JM, Biddle JW, Ferraro MJ, Jacoby GA, Tenover FC. 2000. Characterization of the extended-spectrum beta-lactamase reference strain, *Klebsiella pneumoniae* K6 (ATCC 700603), which produces the novel enzyme SHV-18. Antimicrob Agents Chemother 44:2382–2388. doi:10.1128/AAC.44.9.2382-2388.2000. 10952583 PMC90073

